# Inhibition of cathepsin S confers sensitivity to methyl protodioscin in oral cancer cells via activation of p38 MAPK/JNK signaling pathways

**DOI:** 10.1038/srep45039

**Published:** 2017-03-22

**Authors:** Ming-Ju Hsieh, Chiao-Wen Lin, Mu-Kuan Chen, Su-Yu Chien, Yu-Sheng Lo, Yi-Ching Chuang, Yi-Ting Hsi, Chia-Chieh Lin, Jui-Chieh Chen, Shun-Fa Yang

**Affiliations:** 1 Cancer Research Center, Changhua Christian Hospital, Changhua, 500, Taiwan; 2 School of Optometry, Chung Shan Medical University, Taichung, 40201, Taiwan; 3 Graduate Institute of Biomedical Sciences, China Medical University, Taichung, 404, Taiwan; 4 Institute of Oral Sciences, Chung Shan Medical University, Taichung, 40201, Taiwan; 5 Department of Dentistry, Chung Shan Medical University Hospital, Taichung, 40201, Taiwan; 6 Department of Otorhinolaryngology-Head and Neck Surgery, Changhua Christian Hospital, Changhua, 500, Taiwan; 7 Department of Pharmacy, Changhua Christian Hospital, Changhua, 500, Taiwan; 8 College of Health Sciences, Chang Jung Christian University, Tainan, 71101, Taiwan; 9 Department of Recreation and Holistic Wellness, Mingdao University, Changhua, 52345, Taiwan; 10 Department of Biochemical Science and Technology, National Chiayi University, Chiayi, 600, Taiwan; 11 Institute of Medicine, Chung Shan Medical University, Taichung, 40201, Taiwan; 12 Department of Medical Research, Chung Shan Medical University Hospital, Taichung, Taiwan

## Abstract

Oral cancer is one of the most common cancers in the world. Approximately 90% of oral cancers are subtyped to oral squamous cell carcinoma (OSCC). Despite advances in diagnostic techniques and improvement in treatment modalities, the prognosis remains poor. Therefore, an effective chemotherapy mechanism that enhances tumor sensitivity to chemotherapeutics is urgently needed. Methyl protodioscin (MP) is a furostanol bisglycoside with a wide range of beneficial effects, including anti-inflammatory and anti-cancer properties. The aim of the present study was to determine the antitumor activity of MP on OSCC and its underlying mechanisms. Our results show that treatment of OSCC cells with MP potently inhibited cell viability. Moreover, MP leading to cell cycle arrest at G2/M phase, which subsequently activates caspase-3, -8, -9 and PARP to induce cell apoptosis. Meanwhile, we also demonstrate that MP induces a robust autophagy in OSCC cells. The results indicate cathepsin S (CTSS) is involved in MP-induced apoptosis and autophagy by modulation of p38 MAPK and JNK1/2 pathways. These findings may provide rationale to combine MP with CTSS blockade for the effective treatment of OSCC.

Oral cancer, the most common head and neck cancer, is any cancerous tissue growth located in the oral cavity leading to more than 145,400 human deaths worldwide every year. Oral squamous cell carcinoma (OSCC) accounts for the vast majority of malignancies in the oral cavity^1^,^2^. Conventional treatment of OSCC includes surgery, radiotherapy, and chemotherapy^3^. Although the clinical outcome of OSCC patients has gradually improved in the last decades, the prognosis of patients with advanced-stage disease is still poor, reflecting limited advances in our understanding of pathogenesis of this disorder^4^. This unmet need highlights the necessity to develop novel therapeutic modalities for patients with advanced and resectable OSCC. Natural phytochemicals have received a great attention in drug discovery, which are becoming an emerging field for chemoprevention and chemotherapy in various diseases, including OSCC^5^,^6^,^7^. Methyl protodioscin (MP) is a furostanol bisglycoside isolated from the *Dioscorea collettii var. hypoglauca* (Dioscoreaceae), which is a traditional herbal medicine with anti-inflammatory and anti-tumor properties^8^,^9^. Previous studies have reported that MP induces G2/M cell cycle arrest and apoptosis leading to strong cytotoxicity across diverse cancer types^9^,^10^,^11^,^12^. However, cytotoxic effect of MP and its mechanism of action in OSCC cells are still unknown. In addition, accumulating studies suggest that protease activity is implicated in driving cancer progression via modulating both autophagy and apoptosis^13^,^14^,^15^,^16^. Two major routes of programmed cell death are closely associated with tumor resistance to anticancer drugs. Thus we tested whether proteases is involved in MP-induced cytotoxicity in OSCC cells. Here, we report that Cathepsin S (CTSS), a member of the cysteine cathepsin protease family, is involved in MP-induced cell death. CTSS is a lysosomal enzyme which is overexpressed in various cancer types and can promote lysosomal degradation of a variety of damaged or unwanted proteins^13^,^17^,^18^,^19^. There is increasing evidence to suggest CTSS plays a critical role in the regulation of autophagy and apoptosis^16^,^20^,^21^. In this study, we aim to determine the mechanisms by which MP regulates CTSS levels and subsequently leads to the apoptosis and autophagy in OSCC cells. Our studies clearly demonstrate that the combined use of MP with CTSS inhibitors results in significant synergy in increasing OSCC cell death, which might be a therapeutic approach to improve the prognosis of OSCC patients.

## Materials and Methods

### Chemicals

Methyl protodioscin (purity >98%) was purchased from Santa Cruz Biotechnology (Santa Cruz, CA). The compound was dissolved in dimethyl sulfoxide (DMSO) and stored at −20 °C. Diluted in cell culture medium to the final concentration before use. The final concentration of DMSO for all treatments was consistently less than 0.1%. DAPI dye, propidium iodide (PI), RNase A, protease inhibitor cocktail, phosphatase inhibitor cocktail, 3-(4,5-dimethylthiazol-2-yl)-2,5-diphenyltetrazolium bromide (MTT), acridine orange (AO) and monodansylcadaverine (MDC) were purchased from Sigma-Aldrich (St Louis, MO). Antibody against Cdc2, Cyclin A, Cyclin B1, p21 Cip1, p27 Kip1, cleaved caspase-3, -8, and -9, cleaved poly (ADP-ribose) polymerase (PARP), LC3B, p62, Beclin1, Cathepsin S, p-AKT, AKT, p-p38, p38, p-ERK1/2, ERK1/2, p-JNK1/2, JNK1/2, and GAPDH were purchased from Cell Signaling Technology (Danvers, MA). Specific inhibitors for p38 MAPK inhibitor (SB203580) and JNK inhibitor (SP600125) were purchased from Santa Cruz Biotechnology (Santa Cruz, CA). The commercial Cathepsin S inhibitor Z-FL-COCHO was purchased from Calbiochem (San Diego).

### Cell culture

The human oral squamous cell carcinoma (OSCC) cell lines (SAS and SCC9) were purchased from the American Type Culture Collection (ATCC) (Manassas, VA). SCC9 cells were cultured in Dulbecco’s modified Eagle’s medium-F12 supplemented with 10% fetal bovine serum (FBS), 1% NEAA, 1 mM glutamine, 1% penicillin/streptomycin, 1.5 g/L sodium bicarbonate, 25 mM HEPES (pH 7.4), hydrocrostine (0.4 mg/L), 1 mM sodium pyruvate and 2 mM glutamine (Sigma, St. Louis, Mo, USA). SAS cells were cultured in Dulbecco’s modified Eagle’s medium-F12 supplemented with 10% FBS, 1 mM glutamine, 1% penicillin/streptomycin, 1.5 g/L sodium bicarbonate, 25 mM HEPES (pH 7.4) and 1 mM sodium pyruvate. The cells culture was maintained at 37 °C in a humidified atmosphere of 5% CO_2_.

### Cell cytotoxicity

MTT assay was used to evaluate the effect of MP on cell viability. Briefly, cells were seeded into 96-well plates at a density of 5000 cells/well containing 100 μl of culture medium. After overnight incubation to allow for attachment, the cells were incubated with indicated MP concentration. At every indicated time interval after MP treatment, 10 μl of MTT (5 mg/ml) was added to each well and incubated for further 4 h at 37 °C. The supernatant was then discarded, and 200 μl of DMSO was added to each well to dissolve the formazan crystals. Optical density (OD) was evaluated by measuring the absorbance, with a test wavelength of 490 nm and a reference wavelength of 630 nm.

### Detection of apoptosis by DAPI staining

After being subjected to indicate treatment, cells were collected and fixed with 4% paraformaldehyde for 20 min. Then, cells plated on the slides and stained with DAPI dye (50 μg/ml) for 10 min. After washing with phosphate-buffered saline (PBS), the morphological changes related to apoptosis were assessed by fluorescence microscopy (Lecia, Bensheim, Germany). Percentage of apoptotic cells was scored on at least 500 cells.

### Cell-cycle analysis

Cells were seeded in six-well dishes. After indicated treatments, cells fixed in 70% ethanol at −20 °C for 16 h. After washing with PBS, cells were incubated for 30 min in the dark at room temperature with PI buffer (4 mg/ml PI, 1% Triton X-100, 0.5 mg/ml RNase A in PBS) and then filtered through a 40-mm nylon filter (Falcon, USA). The cell cycle distribution was analyzed by flow cytometry.

### Annexin V/PI double staining

As previously described^22^. A Muse Annexin V & Dead Cell Assay Kit (Millipore) was used to quantify cell number in different stages of cell death. Briefly, 1 × 10^5^ cells were resuspended in 100 μl PBS (2% BSA). Add 100 μl of Muse™ Annexin V & Dead Cell Reagent to each tube; the cell suspension was incubated for 20 min at room temperature in the dark. Analyze by Muse Cell Analyzer flow cytometry and analysis data by the Muse^®^ Cell Analyzer Assays (Millipore).

### Western blot analysis

Cells were harvested and lysed in RIPA buffer containing protease inhibitor cocktail and phosphatase inhibitor cocktail. Protein concentration was determined by the BCA assay (Pierce). The equal amount of total protein was resolved by SDS-PAGE and transferred to PVDF membranes (Millipore, Bedford, MA). Membranes were blocked with 5% non-fat milk in TBST for 1 h and then incubated at 37 °C for 1 h or at 4 °C overnight with the indicated primary antibodies. Membranes were washed with TBST and incubated for 1 h at room temperature with the appropriate secondary antibodies conjugated to horseradish peroxidase. Membranes were then washed and bound antibodies were visualized using a chemiluminescence (ECL) detection kit (Millipore).

### Detection and quantification of autophagic cells by staining with acridine orange (AO) and monodansylcadaverine (MDC)

Cells cultured in 6-well plates were either treated with indicated concentrations of MP or left untreated as control for 24 h at 37 °C. Thereafter, cells were stained with 1 μg/ml AO or 50 μM MDC in fresh medium and incubated for 30 min at 37 °C in the dark. After three times washing with PBS, cells were immediately visualized by a fluorescence microscope.

### Human protease antibody array

Cells were treated with or without MP for 24 h in DMEM medium. Human protease antibody array (R&D Systems) was performed as per the manufacturer’s instruction. Briefly, the cell lysates (600 μg) were mixed with array buffer and incubated with pre-blocked array membrane at 4 °C for overnight. Membranes were then washed and incubated with primary antibody cocktail for 2 h, followed by washing and incubated with secondary antibody for 30 min. Membranes were then washed again and bound antibodies were visualized using ECL reagents and autoradiography.

### Knockdown of CTSS by small interfering RNA (siRNA)

The specific siRNA against CTSS and scramble control siRNA were purchased from Invitrogen. The target sequence of CTSS is 5′-CCACAACUUUGGUGAAGAA-3′. Gene silencing was performed using an RNAiMax reagent (Invitrogen) by following the manufacturer’s instructions. In brief, the cells were seeded onto 6-well culture plates and allowed to grow overnight to reach an appropriate confluence. The siRNA oligonucleotides and RNAiMAX reagent were separately diluted and then mixed. After 20 min of incubation at room temperature, the cells were incubated with the transfection medium and incubated for overnight at 37 °C in a humidified atmosphere of 5% CO_2_.

### Expression of CTSS expression vector in OSCC cells

Full-length human CTSS plasmid (pCMV3-CTSS-His) was amplified by PCR using cDNA of human cells and cloned into the Kpnl and Xbal site of pCMV3-C-His vector (Sequencing primers: T7(TAATACGACTCACTATAGGG), BGH(TAGAAGGCACAGTCGAGG)). The constructed plasmid was transiently transfected into OSCC cells using FuGENE 6 (Roche Diagnostics, Basel, Switzerland). After 24 h of transfection, the cells were used for the following experiments.

### Statistical Analysis

Values represent the means ± standard deviation and the experiments were repeated at least three times. Statistical analyses were performed using the one-way analysis of variance (ANOVA) followed by Tukey’s *post-hoc* test was used when more than three groups were analyzed. Data comparisons were performed with Student’s *t* test (Sigma-Stat 2.0, Jandel Scientific, San Rafael, CA) when two groups were compared. In all cases, a *p* value < 0.05 was considered to be statistically significant.

## Results

### Cytotoxic effects of Methyl protodioscin on human oral cancer cells

**Figure 1:**
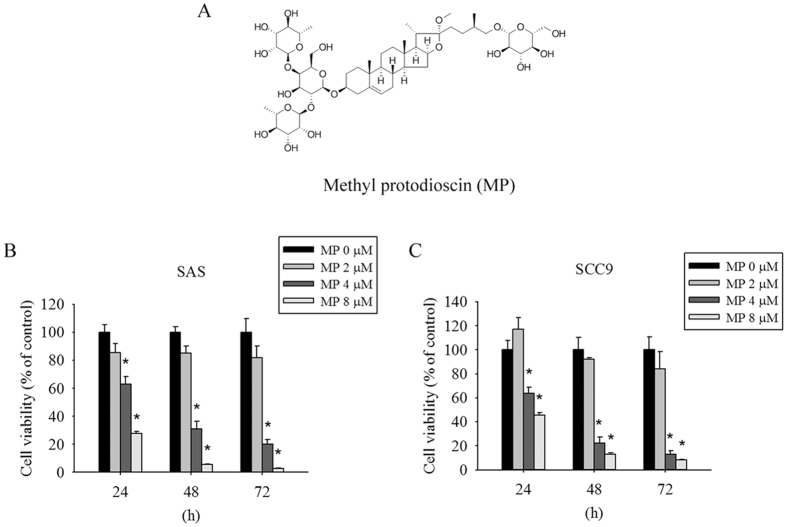
Cytotoxic effects of MP on human oral cancer cells. (**A**) Chemical structure of MP. (**B**) SAS and (**C**) SCC9 cells were treated with increasing doses of MP (0, 2, 4 and 8 μM) for different time points (24, 48 and 72 h). Cell viability was measured by MTT assay. Results are shown as mean ± SD from 3 determinations per condition repeated 3 times. *P < 0.05, compared with the control (0 μM).

The chemical structure of Methyl protodioscin (MP) is shown in Fig. 1A. To examine the effect of MP on cell viability, SAS and SCC9 were treated with different concentrations of MP for 24, 48, and 72 h. As shown in Fig. 1B,C, MP significantly inhibited cell viability in a dose- and time-dependent manner. These results indicate that MP can potently inhibit cell viability in different human OSCC cell lines.

### MP-induced cell apoptosis and G2/M arrest in human OSCC cell lines

**Figure 2:**
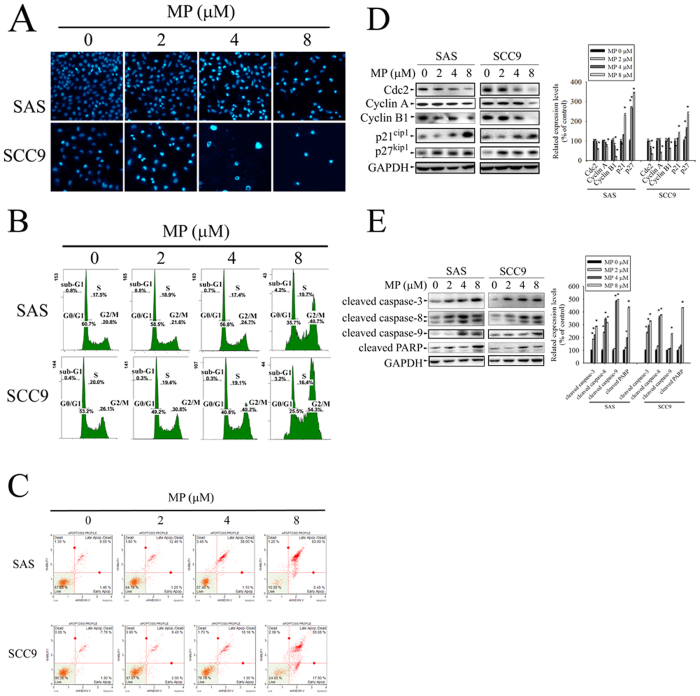
MP induces apoptosis and G2/M phase cell cycle arrest in SAS and SCC9 cells. Cells were treated with different concentration of MP (0, 2, 4 and 8 μM) for 24 h. (**A**) Morphological changes in cells including nuclei condensation and fragmentation were observed by DAPI staining under a fluorescence microscope. (**B**) Cell cycle was analysed by PI staining and flow cytometry. Sub-G1, G0/G1, S and G2/M indicate different cell cycle phases. (**C**) Apoptotic cells were stained with Annexin V-FITC/PI and analyzed by flow cytometry. (**D**) The left panel shows representative Western blots for the effect of MP on expression of cell cycle regulatory proteins (Cdc2, Cyclin A, Cyclin B1, p21 Cip1, and p27 Kip1). Bar graphs represent the relative density of each band normalized to GAPDH (right panel). (**E**) The left panel shows representative Western blots for the effect of MP on expression of apoptosis-related proteins (cleaved caspase-3, -8, -9 and cleaved PARP). Bar graphs represent the relative density of each band normalized to GAPDH (right panel). Values represent the mean ± SD of 3 independent experiments. *P < 0.05, compared with the control (0 μM).

To determine whether MP inhibits cell growth through the induction of apoptosis, DAPI staining was used to test cell apoptosis in OSCC cell lines. As shown in Fig. 2A, apoptotic cells with condensed chromatin were gradually increased in a dose-dependent fashion. The cell cycle distribution was also determined by PI staining and flow cytometry after exposing various doses of MP. As shown in Fig. 2B, MP induced a significant increase in accumulation of G2/M population of cells in a dose dependent manner. To further confirm that MP induces cell apoptosis, cells were stained with annexin V-FITC and PI, and subsequently analyzed by flow cytometry. The results revealed that MP induced a dose-dependent increase in the percentage of both early and late apoptotic cells (Fig. 2C). To understand the mechanisms by which MP induces G2/M phase arrest and apoptosis to inhibit cell growth, we next examined the effect of MP on the expression of key cell cycle regulators (Cdc2, Cyclin A, Cyclin B1, p21 Cip1, and p27 Kip1) and apoptosis-related proteins (cleaved caspase-3, -8, -9 and cleaved PARP) in SAS and SCC9 cells by Western blot. We observed variable alterations in the expression levels of cell cycle regulators (Fig. 2D). The expression levels of Cdc2, Cyclin A, and Cyclin B1 were significantly decreased in a concentration-dependent manner. In contrast, there was a dose-dependent increase in the expression level of negative regulators (p21 Cip1, and p27 Kip1) of cell cycle progression. Furthermore, MP also caused a dramatic dose-dependent increase in the protein level of cleaved caspase-3, -8, and 9 and cleaved PARP (Fig. 2E). These results indicate that MP is able to decrease the level of Cdc2, Cyclin A, and Cyclin B1 and to increase the level of p21Cip1 and p27Kip1 simultaneously resulting in cell cycle arrest at G2/M phase, which subsequently leads to apoptotic cell death through cleavage of caspase-3, -8, -9 and PARP.

### MP induced autophagy in human OSCC cell lines

**Figure 3:**
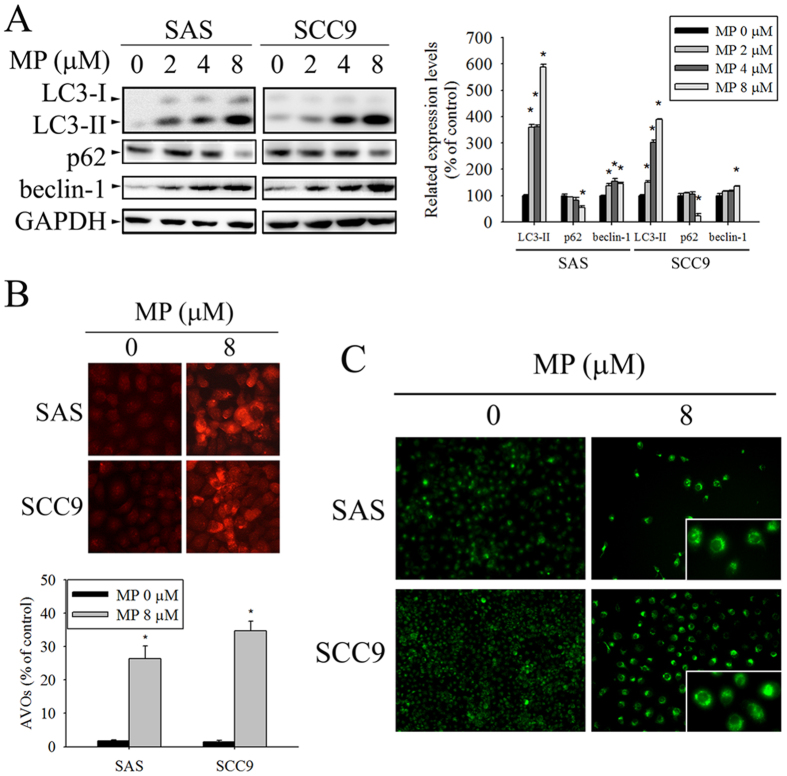
MP induces autophagy in SAS and SCC9 cells. (**A**) Cells were treated with indicated concentration of MP for 24 h and the expression levels of LC3-I/LC3-II, p62, and beclin-1 were examined by Western blot (left panel). GAPDH was used as an internal control to normalize the amount of proteins applied in each lane (right panel). (**B**) Cells were treated with DMSO (control) or 8 μM MP for 24 h, and then were stained with acridine orange (AO) for acidic vesicular organelles (AVOs) formation. An increase in number of cells with accumulating AVOs (orange-red fluorescence) was examined under a fluorescence microscope. The orange-red punctate spots were considered to be AVOs, markers for autophagosomes. The quantification of cell with AVOs was performed using the analyze particles tool of the Image J software on an average of 100 cells (lower panel). (**C**) Cells were treated with DMSO (control) or 8 μM MP for 24 h, and MDC was added to the medium during the last hour of culture. MDC-stained cells are indication of autophagosome formation. The data show the mean ± SD of at least 3 independent experiments. *P < 0.05, compared with the control (0 μM).

Autophagy was proved to mediate cell death under specific circumstances, which can also be accompanied by apoptosis. Thus, we also examined whether or not MP induces autophagy in OSCC cells through biochemical and morphological analyses. As shown in Fig. 3A, we analyzed the expression levels of three autophagy-related proteins, namely LC3I/II, Beclin-1, and p62 by Western blot. The results showed that MP increased the protein levels of LC3I/II and beclin-1, while P62 was decreased in a dose-dependent manner, indicating marked induction of autophagy. To further confirm MP-induced autophagy, we examined the formation of autophagic vacuoles using the specific fluorescent dyes AO and MDC. MP treatment resulted in an increased formation of AVOs in SAS and SCC9 cells using fluorescence microscopy upon staining with the lysosomotropic agent AO (Fig. 3B). Untreated cells showed very little cytoplasmic red staining corresponding to lysosomes; however, MP-treated cells displayed many spots of red fluorescence, indicating the formation and accumulation of acidic vesicles. Consistent with AO staining, the green fluorescence intensities of MDC-labeled autophagic vacuoles were markedly increased in SAS and SCC9 cells after exposure to MP (Fig. 3C). Overall, these results clearly indicate that MP induces a robust autophagy in OSCC cells.

### CTSS expression was down-regulated after treatment with MP involved in the cytotoxic effects

**Figure 4:**
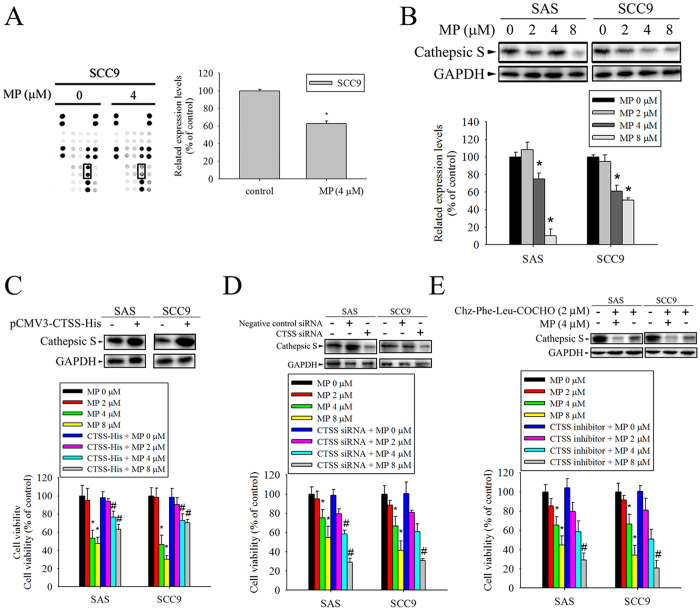
Cathepsin S (CTSS) is required for MP-induced cell death in SAS and SCC9 cells. (**A**) Template shows the location of specific antibodies spotted onto the human protease array kit. Each antibody was spotted in duplicate. A result reveals the difference between SCC9 cells treated with or without MP. Right panel is the result of densitometry data of the pair of duplicate spots representing a marked protein (CTSS). (**B**) Cells were incubated with various concentrations of MP for 24 h. The protein expression levels of CTSS were examined by Western blot (upper panel). The intensity of bands was quantified by densitometry analysis (lower panel). (**C**) CTSS -expressing vector was transfected into cells, followed by analysis of CTSS expression using Western blot (upper panel). GAPDH was used as the internal protein loading control. Overexpression of CTSS induces MP resistance in cells (lower panel). (**D**) Cells were transiently transfected with CTSS siRNA or control siRNA, followed by analysis of CTSS expression using Western blot (upper panel). Silencing of CTSS in cells resulted in increased MP sensitivity (lower panel). (**E**) Cells were treatment with CTSS inhibitor (Chz-Phe-Leu-COCHO, 2 μM)), followed by analysis of CTSS expression using Western blot (upper panel). The effect of the CTSS inhibitor on cell viability after treated with MP. Inhibition of CTSS activity caused an increased in MP sensitivity (lower panel). The cell viability was assessed by MTT assay and indicated as a percentage of the values measured at each dose point. Values represent the mean ± SD from 3 determinations per condition repeated 3 times. *P < 0.05, compared with the control (0 μM), control siRNA, and DMSO control. ^#^*P* < 0.05, compared with MP (8 μM), respectively.

Accumulating evidence reveals that proteases have a potent role in modulating apoptosis and autophagy, which might be regulators of cancer progression and therapeutic response^14^,^15^,^23^. To assess the function of proteases, a protease array was used to screen for expressed levels of diverse proteases following stimulation of SCC9 cells with MP, compared with vehicle. As shown in Fig. 4A, the expressed level of CTSS was decreased under MP treatment. To confirm the effect of MP on CTSS expression, SAS and SCC9 cells were cultured in the presence of increasing concentrations of MP for 24 h. The results showed that MP suppressed CTSS expression in a concentration-dependent manner (Fig. 4B). Furthermore, we also investigated whether overexpression or knockdown of CTSS in cells would mediate MP sensitivity. The results reveal that overexpression of CTSS could render cells more resistant to MP (Fig. 4C); while knockdown of CTSS could be sensitive to MP (Fig. 4D). In addition, the inhibition of CTSS activity by inhibitor was also significantly increase MP sensitivity (Fig. 4E). These findings suggest that CTSS is involved in MP-induced cell death.

### Effect of CTSS on MP-induced apoptosis and autophagy

**Figure 5:**
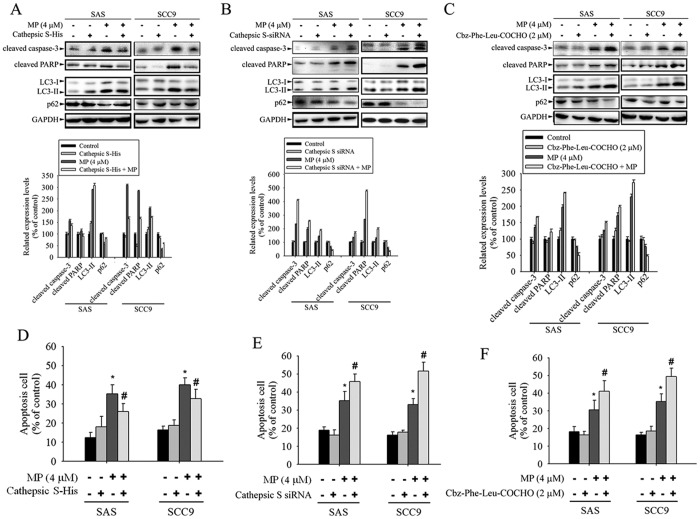
CTSS is involved in MP-induced apoptosis and autophagy in SAS and SCC9 cells. (**A**) Cells transfected with or without CTSS expressing plasmid for 24 h were either untreated or treated with MP (4 μM) for another 24 h. (**B**) Cells transfected with or without CTSS siRNA for 24 h were either untreated or treated with MP (4 μM) for another 24 h. (**C**) Cells were pretreated with the indicated inhibitors for 4 h and then cultured with or without MP (4 μM) for 24 h. Equivalent amounts of total cell lysates were subjected to Western blot with the indicated antibodies (cleaved caspase-3, cleaved PARP, LC3-I/LC3-II, and p62). The relative density of the bands was quantified by densitometry analysis. Data are presented after normalization with the GAPDH bands. (Bottom panel) (**D**–**F**) The percentages of apoptotic cells were evaluated for apoptosis cell content using flow cytometry. Values represent the mean ± SD of 3 independent experiments. *P < 0.05, compared with the control (cells treated with DMSO only). ^#^*P* < 0.05, compared with cells treated with MP (4 μM) alone.

We next investigated whether CTSS is involved in MP-induced apoptosis and autophagy in SAS and SCC9 cells. The involvement of apoptosis-related proteins (cleaved caspase-3 and cleaved PARP) and autophagy-related proteins (LC3 I/II and p62) were determined by Western blot analysis. As shown in Fig. 5A, overexpression of CTSS markedly attenuated MP-induced increase in the amount of cleaved caspase-3, cleaved PARP, and LC3 I/II but decrease in p62 expression. In contrast, knockdown of CTSS and inhibition of its enzymatic activity were significantly enhanced MP-induced apoptosis and autophagy as indicated by increased caspase-3 and PARP cleavage; LC3-II elevation and p62 degradation (Fig. 5B,C). We then evaluated the role of CTSS in MP-induced apoptosis by PI staining. As expected, overexpression of CTSS prevents MP-induced death, but CTSS depletion induces significant increase of the apoptosis cell population, as assessed by flow cytometry (Fig. 5D–F). Taken together, these results suggest that inhibition of CTSS is able to promote a synergistic cytotoxic effect with MP, leading to induction of both apoptotic and autophagic cell death.

### P38 MAPK/JNK signaling pathways are involved in the effect of CTSS on MP-induced apoptosis and autophagy

**Figure 6:**
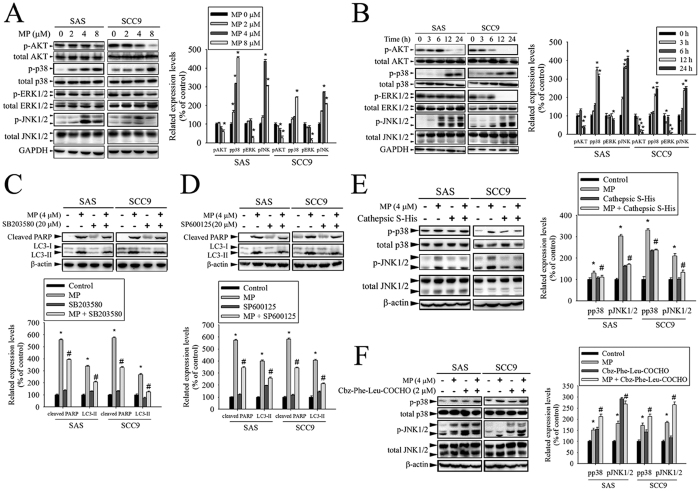
CTSS blocked the effects of MP on p38 MAPK and JNK1/2 in SAS and SCC9 cells. (**A**) Cells were treated with different concentrations of MP (0–8 μM) for 24 h. The p-AKT, p-p38, p-ERK1/2, and p-JNK1/2 were analyzed by Western blot with their respective antibodies. The total protein of AKT, p38, ERK1/2, and JNK1/2 is also shown. The intensity of the phosphorylation signals was determined by densitometry and normalized to their total protein levels (right panel). (**B**) Cells were treated with MP (8 μM) for indicated time intervals and the phosphorylation status and total levels of proteins were measured by Western blot. Quantitative analyses of the phosphorylation status and total levels of proteins are shown in the right panel. (**C**,**D**) Effects of the inhibition of p38 MAPK and JNK1/2 on MP-induced apoptosis and autophagy were assessed by Western blot using specific antibodies (cleaved PARP and LC3-I/LC3-II). Cells were pretreated with SB203580 (p38 MAPK inhibitor, 10 μM) or SP600125 (JNK inhibitor, 20 μM) for 1 h followed by treatment with or without MP for 24 h. Band intensity was quantified by densitometry analysis. β-actin was used as an internal control to normalize the amount of proteins (Bottom panel). (**E**,**F**) The effects of CTSS overexpression or CTSS inhibitor on MP-induced activation of p38 MAPK and JNK1/2 were assessed by Western blot. Quantitative analyses of the phosphorylation status and total levels of proteins are shown in the right panel. Values represent the mean ± SD of 3 independent experiments. *P < 0.05 vs. control (cells treated with DMSO only), and ^#^*P* < 0.05 vs. MP.

Previously studies have reported that AKT and MAPKs signaling pathways are implicated in apoptosis and autophagy^24^,^25^,^26^,^27^. To further investigate the mechanism by which MP induces apoptosis and autophagy, we directly measured the phosphorylation of AKT and MAPKs in response to MP. The results revealed that stimulation of cells to MP induced an increase in phosphorylation of p38 MAPK and JNK in a dose- and time-dependent manner (Fig. 6A,B). To further clarify whether MP-induced apoptosis and autophagy depended on p38 MAPK/JNK signaling pathways, cells were pre-treated with p38 MAPK inhibitor (SB203580) or JNK inhibitor (SP600125) for 1 h followed by treatment with or without MP for 24 h. The results showed that cleaved PARP and LC3-II were slightly decreased in MP co-treatment with SB203580 or SP600125 group compared with MP treatment alone (Fig. 6C,D). In addition, we further show that overexpression of CTSS blocked MP-induced activation of p38 MAPK and JNK (Fig. 6E). However, inhibition of CTSS activity resulted in significant enhancement of MP-induced activation of p38 MAPK and JNK (Fig. 6F). Collectively, these data indicated that the inhibition of CTSS led to sensitization of OSCC cells to MP cytotoxicity, which might be related to the activation of p38 MAPK/JNK signaling pathways.

## Discussion

MP has been previously tested by NCI’s (National Cancer Institute, USA) anti-cancer drug screen on a panel of 60 human tumor cell lines. The results showed that MP had strong cytotoxic activity and might have a novel mechanism of anti-cancer action^9^. However, OSCC cells was not included in the NCI’s 60 cell lines, the bioactivity of MP on a human oral cancer cells requires further research. In this study, we investigated whether MP exert a strong cytotoxicity against human oral cancer cells. Here, our results indicate for the first time that MP can significantly repress the expression of CTSS leading to inducing autophagy and subsequent apoptosis in human OSCC cells. Furthermore, we found that the CTSS is involved in ROS-mediated p38 MAPK/JNK signaling pathways in the regulation of autophagy and apoptosis in MP-treated cells.

Accumulating evidence suggests that MP, a furostanol saponin with four sugar units, has strong anti-tumor properties. In human chronic myelogenous leukemia cells, a study showed that MP inhibits cell proliferation via G2/M arrest and apoptosis, which are attributed to down-regulation of cyclin B1, Ca^2+^ homeostatic perturbation, mitochondrial dysfunction, and ROS generation^12^. Anticancer effects of MP with similar mechanisms of action have also been observed in human liver and lung cancer cells^10^,^11^. In addition to anti-cancer activities, MP has been recommended for the treatment of inflammation in the airway and intestine through modulation of immune responses^8^,^28^. Recently, inflammation has been considered as a key underlying mechanism for cancer development^29^. Notably, most anti-inflammatory compounds are potent anti-cancer agents. More importantly, several studies indicate MP is relatively safe without any side effects, suggesting that MP might serve as therapeutic approach for controlling oral cancer^30^,^31^.

In the present study, we demonstrated that inhibiting CTSS in OSCC cells can enhance MP-induced generation of ROS. Growing evidence in recent years suggests that ROS are involved in stimulation of autophagy during cancer initiation and progression^32^,^33^. Autophagy is self-digestion process that degrades intracellular long-lived proteins and damaged organelles in response to stress, which is implicated in both cell survival and death. Numerous reports have shown that extensive or inappropriate activation of autophagy can lead to apoptotic cell death^34^,^35^. Moreover, many anticancer therapies are able to increase ROS-induced autophagy leading to cell death^36^,^37^.

Autophagy is a dynamic multi-step process that involves fusion of the autophagosome with the lysosome to form the autolysosome, which have been shown to have an acidic pH and contain various proteases^38^. CTSS is a lysosomal protease that can promote degradation of proteins in the autolysosome^39^. Several studies have shown that CTSS plays a critical role in the regulation of autophagy and apoptosis^16^,^40^,^41^. In addition, CTSS has been shown to be highly expressed in a variety of cancers including brain^42^,^43^, lung^44^, gastric^45^,^46^, colorectal^19^,^47^, hepatocellular^48^, and prostate^49^,^50^ carcinomas, which might involve in clinical aggressiveness.

CTSS is therefore an attractive target for cancer therapy to prevent disease progression. Previous studies have demonstrated CTSS knockout mice causes reduced tumor vascularization and tumor growth^18^,^51^,^52^. These results indicate that the inhibition of CTSS activities may have clinical utility to abrogate tumor development. Over the last few years, inhibition of CTSS by a chemical inhibitor, RNA interference, or antibody has shown that targeting CTSS exhibits antitumor activity using *in vitro* and *in vivo* tumor models^16^,^20^,^47^,^53^,^54^,^55^,^56^. More recently, a study further demonstrated that a combination of the CTSS inhibitor and the EGFR tyrosine kinase inhibitor markedly promoted tumor apoptosis, indicating that CTSS is a potential novel therapeutic target for cancer treatment^57^.

In conclusion, our results reveal that MP can reduce CTSS expressed level to induce cell cycle arrest, autophagy, and apoptosis in human oral cell lines through mediated p38 MAPK/JNK signaling pathways, suggesting that MP and CTSS are attractive candidates for tumor therapies. We believe that MP and CTSS may promise candidates for development of antitumor drugs targeting oral cancer.

## Additional Information

**How to cite this article**: Hsieh, M.-J. *et al*. Inhibition of cathepsin S confers sensitivity to methyl protodioscin in oral cancer cells via activation of p38 MAPK/JNK signaling pathways. *Sci. Rep.* **7**, 45039; doi: 10.1038/srep45039 (2017).

**Publisher's note:** Springer Nature remains neutral with regard to jurisdictional claims in published maps and institutional affiliations.
